# Spectral Radiation Dependent Photoprotective Mechanism in the Diatom *Pseudo-nitzschia multistriata*

**DOI:** 10.1371/journal.pone.0087015

**Published:** 2014-01-24

**Authors:** Christophe Brunet, Raghu Chandrasekaran, Lucia Barra, Vasco Giovagnetti, Federico Corato, Alexander V. Ruban

**Affiliations:** 1 Laboratory of Ecology and Evolution of Plankton, Stazione Zoologica Anton Dohrn, Villa Comunale, Napoli, Italy; 2 School of Biological and Chemical Sciences, Queen Mary University of London, Mile End Road, London, United Kingdom; Stazione Zoologica, Italy

## Abstract

Phytoplankton, such as diatoms, experience great variations of photon flux density (PFD) and light spectrum along the marine water column. Diatoms have developed some rapidly-regulated photoprotective mechanisms, such as the xanthophyll cycle activation (XC) and the non-photochemical chlorophyll fluorescence quenching (NPQ), to protect themselves from photooxidative damages caused by excess PFD. In this study, we investigate the role of blue fluence rate in combination with red radiation in shaping photoacclimative and protective responses in the coastal diatom *Pseudo-nitzschia multistriata*. This diatom was acclimated to four spectral light conditions (blue, red, blue-red, blue-red-green), each of them provided with low and high PFD. Our results reveal that the increase in the XC pool size and the amplitude of NPQ is determined by the blue fluence rate experienced by cells, while cells require sensing red radiation to allow the development of these processes. Variations in the light spectrum and in the blue versus red radiation modulate either the photoprotective capacity, such as the activation of the diadinoxanthin-diatoxanthin xanthophyll cycle, the diadinoxanthin de-epoxidation rate and the capacity of non-photochemical quenching, or the pigment composition of this diatom. We propose that spectral composition of light has a key role on the ability of diatoms to finely balance light harvesting and photoprotective capacity.

## Introduction

**Figure 1:**
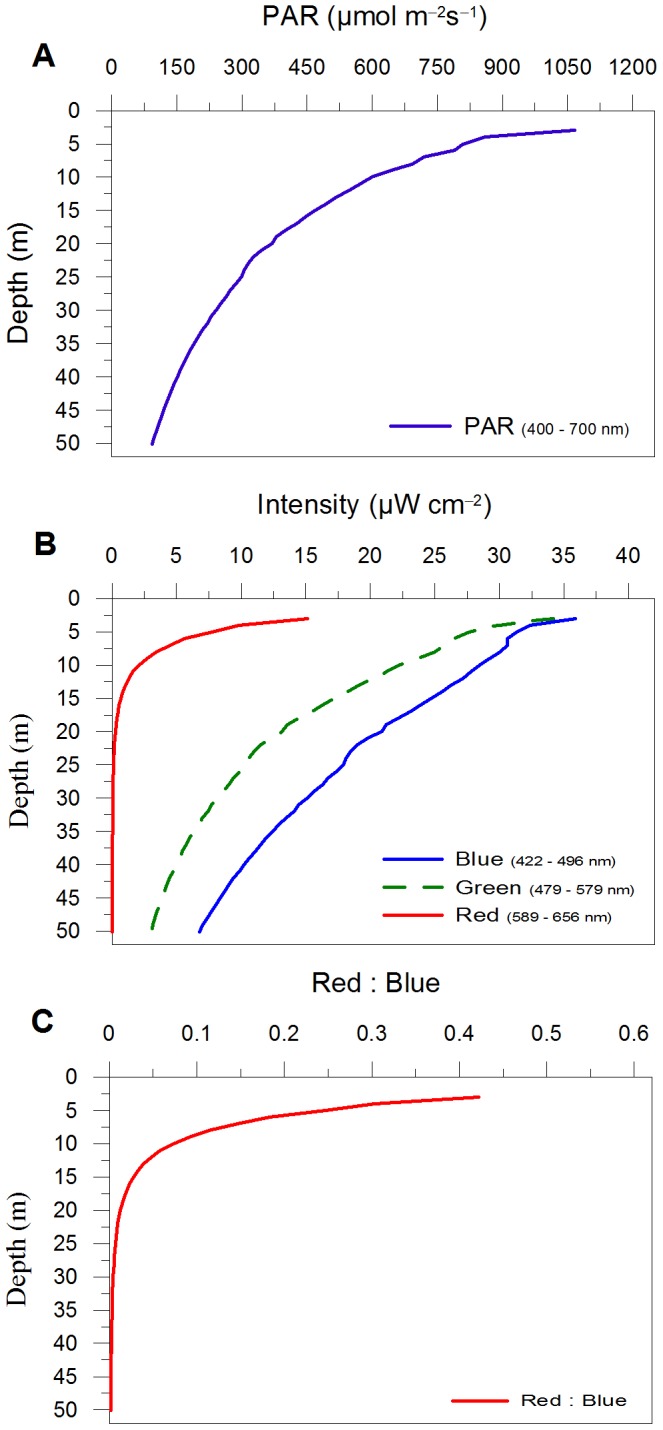
*In situ* light profile *vs* depth. (A) PAR (µmol photons m^–2^ s^–1^) distribution along the water column, (B) blue, red and green radiation distribution along the water column (µW cm^–2^; blue –422 to 496 nm, green –479 to 579 nm and red –589 to 656 nm), and (C) distribution of red : blue ratio along the water column. Data are mean of profiles done on 5 stations in the Mediterranean Sea in June–July 2008 (C. Brunet, unpublished data).

Originating some 2.32−2.45 Gyr ago, oxygenic photosynthesis spread across the Earth, allowing the great diversification of life and globally altering the community structure and ecological function of terrestrial and aquatic habitats [1], [2]. Phytoplankton, small floating photosynthetic microorganisms that populate the aquatic realms, thrive in a light environment naturally variable over spatial and temporal extremes [3], [4]. While penetrating through the water column, light intensity (photon flux density; PFD) exponentially decreases (Fig. 1A) due to absorption and scattering by dissolved substances and suspended particles [5]. The unpredictable passing of clouds, motion of waves and turbulent mixing, superimposed to long-term diel and seasonal periodicity, create very complex patterns of short-term fluctuations in the instantaneously available light that controls phytoplankton photosynthesis [6]. In response to such a heterogeneous light environment, phytoplankton have evolved protective mechanisms to harvest light in conditions of excess detrimental PFD, and minimize photo-oxidative damage caused by the formation of reactive oxygen species in the photosystems [7]–[9]. The xanthophyll cycle (XC) and non-photochemical quenching (NPQ) are crucial photoprotective processes that are rapidly activated (seconds to minutes) to dissipate excess absorbed light energy and ensure efficient light harvesting in the photosynthetic membrane [10]–[12].

Together with the PFD variations along the water column, light spectrum also changes greatly, with different attenuation of red, green and blue lights (Fig. 1B), with a steep decrease of the red : blue ratio over the surface layer (Fig. 1C). Interest concerning how does phytoplankton vary its physiological properties with spectral radiation (e.g., [13]–[17]) is still open and, recently, moves towards the intriguing question on how do photosynthetic cells acclimate to and sense the marine light radiation [18]–[23]. Some ecologically-relevant processes, such as aggregation in the dinophyte *Gonyaulax* [24], enhancement of sinking rate [25], and cell motility in diatoms [26] are shown to be triggered by red light, while little information is still available on red light photoreceptors [20]. By contrast, many key physiological processes in algae, such as photomorphogenesis [23], chloroplast movements [27] and cell division in diatoms [23], [28] are triggered by blue light. Blue light photoreceptors, belonging to the aureochrome family, identified in the xanthophyte *Vaucheria frigida* [18] have been shown to be only present in photosynthetic stramenophiles [29]. Very recently, by acclimating *Phaeodactylum tricornutum* to different spectral radiation, Schellenberger Costa et al. showed that blue light perception by aureochrome governs the acclimation and protection processes [21], [22].

The general objective of our study is to decipher the role of spectral radiation on the photophysiological acclimation properties of coastal diatoms, well-known for their high photosynthetic flexibility and regulative capacity [7], [9], [10]. We choose the toxic *Pseudo-nitzschia multistriata* species, since the strain has been recently isolated from the Gulf of Naples (coastal area of the Mediterranean sea, Stazione Zoologica Anton Dohrn, Italy, strain number SY717) and its ecological properties are known (e.g., [30]).

We investigate if and how spectral radiation does affect the photoprotective capacity of this diatom, focussing on the regulation of pigment content, and on the rapidly activated protective responses, as the XC and NPQ. Since the different distribution over water column of the red and blue radiations, and their essential eco-physiological roles (as introduced before), we aim to test the hypothesis that photoprotective capacity in diatoms differs between different mixtures of blue and red radiations, compared to the same radiations when provided separately. We also address the question on the biological effect of the red : blue ratio of the light experienced by cells, as trigger for the photoprotective response ([22]).

The results suggest that spectral composition of light has a key role, together with PFD, on the ability of diatoms to finely balance light harvesting and photoprotective capacity. To our knowledge this is the first report demonstrating the dependence of the XC and NPQ to both the blue and red radiation together.

## Materials and Methods

### Ethics Statement

No specific permits or permissions were required for the field studies, as the cruise for measuring the vertical light profiles was carried out in international waters and the isolation of *Pseudo-nitzschia multistriata* strain SY717 has been done during the long term research Mare-Chiara program in the coastal area of the Gulf of Naples where no specific permits or permissions were required. This work did not involve endangered or protected species.

### Experimental Strategy and Sampling

Four spectral light conditions – blue, red and two mixed light conditions, namely blue-red-green and blue-red were applied (Table 1). The two mixed light conditions were characterized by (i) the same photon flux density (PFD) and relative proportion of red radiation provided (18–20%), and (ii) two different red : blue ratios: 0.43 (blue-red-green) and 0.25 (blue-red), determined by the presence or absence of green light (Table 1). These two values of red : blue ratio characterize the high light environment, 2 m (∼0.43) and 6 m (∼0.25) depths, of the water column during summer in the Mediterranean Sea (Fig. 1).

**Table 1:** Light condition characteristics, and photosynthetic and biochemical properties in *Pseudo-nitzschia multistriata* cells.

	Blue		Blue-red		Blue-red-green		Red
	Low	High	Low	High	Low	High	Low
	(B-L)	(B-H)	(BR-L)	(BR-H)	(BRG-L)	(BRG-H)	(R-L)
PFD	250	450	250	450	250	450	250
Blue	250	450	200	360	105	189	0
Green	0	0	0	0	100	180	0
Red	0	0	50	90	45	81	250
Red : Blue	0	0	0.25	0.25	0.43	0.43	0
a*	2.51 (0.09)	1.46 (0.07)	2.69 (0.22)	2.89 (0.10)	1.97 (0.63)	1.22 (0.08)	4.44 (0.05)
PUR	2.47 (0.09)	2.4 (0.06)	2.27 (0.23)	4.39 (0.16)	1.49 (0.51)	1.95 (0.12)	1.11 (0.23)
_rel_ETR_max_	2.72 (0.13)	1.66 (0.11)	3.61 (0.41)	3.45 (0.17)	2.13 (0.57)	1.07 (0.09)	2.63 (0.47)
α	6.7 (1.1)	3.8 (0.27)	7.3 (0.36)	7.1 (0.44)	4.9 (0.96)	3.0 (0.54)	13 (2.2)
E*k*	408 (66)	431 (21)	497 (32)	491 (39)	472 (37)	363 (84)	200 (5)
POC	131 (37)	62 (8)	80 (5)	80 (5)	100 (8)	89 (5)	154 (28)
POC/PON	4.9 (0.52)	4.3 (0.63)	4.8 (0.27)	4.7 (0.37)	6.2 (0.76)	4.7 (0.05)	2.9 (0.19)
Chl *a*/POC	8.2 (4.8)	10.3 (2.9)	13.8 (2.6)	10.7 (1.5)	9.8 (2.2)	10.8 (0.9)	6.2 (2.5)

Blue, green and red fluence rates (µmol photon m^−2^ s^−1^) measured at light peak and red : blue ratio values for the different light conditions. a*×10^–11^, absorption coefficient (m^2^ cell^−1^); PUR×10^–6^, photosynthetically usable radiation (µW cell^–1^); _rel_ETR_max_×10^–6^, (maximal relative rate of linear electron transport, nmol e^−1^ s^−1^ cell^−1^), α×10^–9^ (maximum light use efficiency, nmol e^−1^ s^−1^ cell^−1^(µmol photon m^−2^ s^−1^)^ −1^), and E*k* (light intensity for reaching _rel_ETR_max_, µmol photon m^−2^ s^−1^); POC, particulate organic carbon (pg cell^−1^); POC/PON, particulate organic carbon (POC) to particulate organic nitrogen (PON) ratio (pg/pg); Chl *a*/POC×10^–3^, Chlorophyll *a* to POC ratio (pg/pg). Data represent mean and standard deviation. For a* and PUR, *n* = 3; For _rel_ETR_max_, α and E*k*, *n* = 6 (mean of the two days light peak measurements); For POC, PON, POC/PON and Chl *a*/POC, *n* = 21.

For each condition, the daily light dose was kept constant, in order to be comparable for the provided photon flux density. Two daily light doses, 6.1 mol m^–2^ d^–1^ and 11 mol m^–2^ d^–1^ (sinusoidal light distribution, peaking at 250 and 450 µmol photons m^–2^ s^–1^, respectively; Table 1), have been tested, with a 12∶12 hours light:dark photoperiod. Light intensity was measured inside each flask by using a laboratory PAR 4 π sensor (QSL 2101, Biospherical Instruments Inc., San Diego, CA, USA), while spectral composition (PAR(λ)) were measured at light peak by using a radiometer (Hyper OCR I, Satlantic, Halifax, CA).

**Figure 2:**
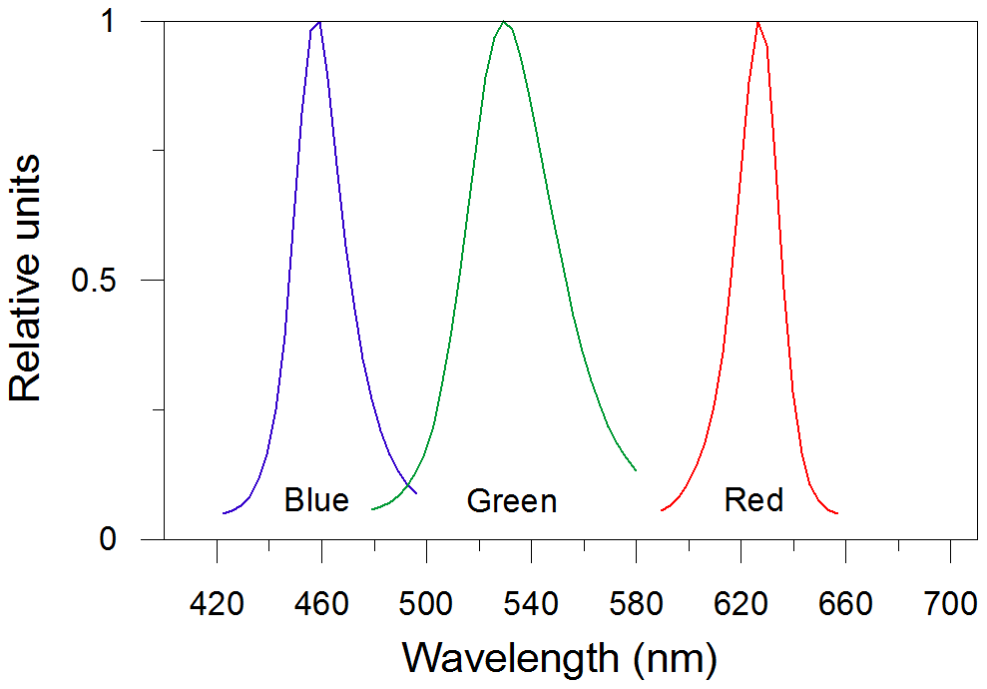
Spectral properties of the LEDs. Blue (422−496 nm), green (480−580 nm) and red light (590−656 nm).

**Figure 3:**
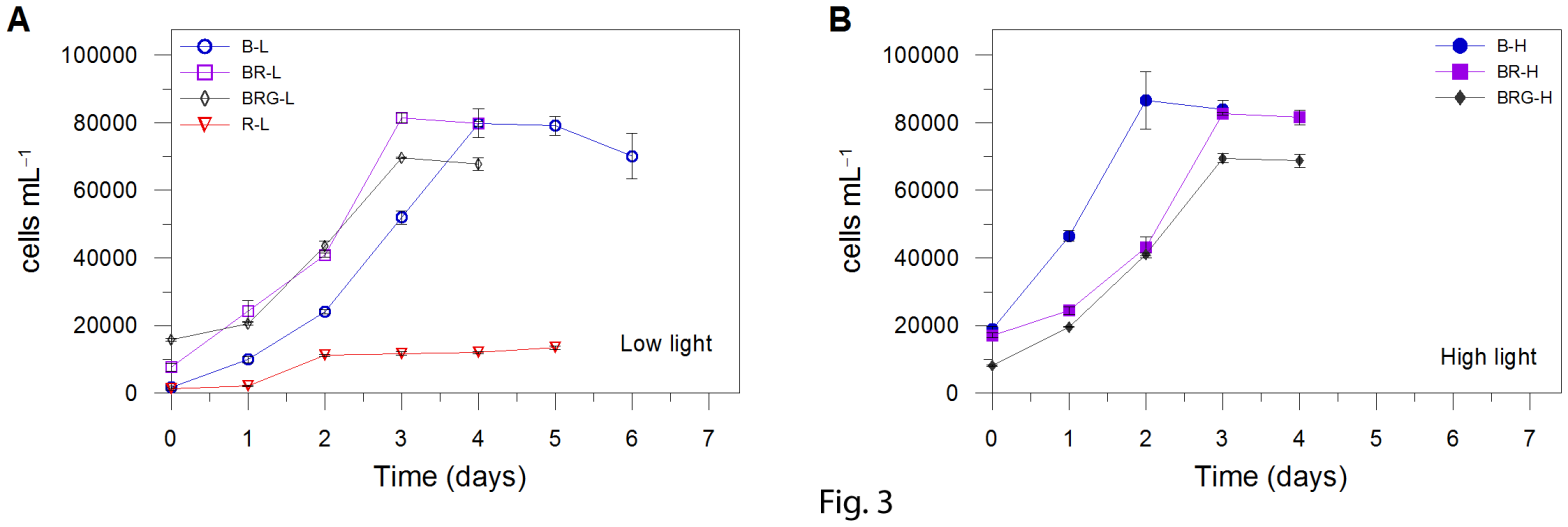
Growth curve of *Pseudo-nitzschia multistriata*. Growth under (A) low and (B) high light. B-L, BR-L, BRG-L, R-L are blue, blue-red, blue-red-green, and red low light conditions, respectively; B-H, BR-H, and BRG-H are blue, blue-red, blue-red-green high light conditions, respectively. Red high light prevented cell growth. Experiments were performed during the exponential phase on days 3 to 5 (B-L), 1 to 3 (R-L, BR-L, BRG-L, B-H) and 2 to 4 (BR-H, BRG-H). Data represent mean ± SD (*n* = 3).

Light was provided by a custom-built illumination system, which allows to monitor and regulate the light intensity and quality. The system is composed by blue, green and red light emitting diodes (peaking at 460, 530 and 626 nm, respectively; Fig. 2). Experiments were conducted on *Pseudo-nitzschia multistriata* strain SY717 isolated in the Gulf of Naples (40° 48′ N, 14° 15′ E, Mediterranean Sea). Cells were cultivated at 20°C in 75 cm^2^ polystyrene canted neck flasks (Corning® flask, Corning Inc., NY, USA), containing natural sterile seawater amended with f/2 nutrients. All the experiments, lasting three days, were performed in triplicate during the exponential growth phase (Fig. 3), on cultures pre-acclimated to each experimental light condition for two weeks before the experiments. Under red light, at high PFD (450 µmol photons m^–2^ s^–1^) cells did not grow, preventing any experimental result.

Samples for pigments, variable fluorescence and electron transport rate, and elemental composition analysis were taken at dawn (time 0), midday (time 6 hours) and in the afternoon (time 9 hours), during the first two days of the experiment, and once (time 0) during the third day. Cell counts were performed daily at time 0, while the absorption spectrum were analysed once per experimental condition (time 6 hours on the second day).

### Cell Concentration

Cell concentration was estimated on triplicate sub-samples. An aliquot of 1 mL was used to fill a Sedgewick Rafter counting cell chamber, and cell counts were performed using a Zeiss Axioskop 2 Plus microscope.

### Photochemical Efficiency and Photosynthetic Parameters

Photochemical efficiency of photosystem (PS) II was estimated by a Phyto-PAM fluorometer (Heinz Walz, Effeltrich, Germany). The variable fluorescence analysis was performed on 15-minutes dark-acclimated samples, to measure the maximum photochemical efficiency (Fv/Fm, [31]). Fm was measured after a saturating pulse of red light (2400 µmol m^–2^ s^–1^, lasting 450 ms), causing a complete reduction of the PSII acceptor pool.

Electron transport rate (ETR) *versus* irradiance curves were determined applying 13 increasing red actinic lights (655 nm) from 1 to 853 µmol photons m^−2^ s^−1^ lasting 1 minute each. The relative electron transport rate (_rel_ETR_,_ expressed in µmol e^–1 ^s^−1^ cell^−1^) was calculated as follows: _rel_ETR = (Fv′/Fm′) · I · 0.5 ·a*.

where, I is the incident irradiance (expressed in µmol photons m^−2^ s^−1^), Fv′ and Fm′ are the variable PS II fluorescence yield and maximal PS II fluorescence yield, respectively, for illuminated cells (measured at the end of the 1 min lasting actinic light), a* is the cell-specific absorption coefficient, expressed in m^2^ cell^−1^ (for the determination of a* see below). A factor of 0.5 was applied to correct for the partitioning of photons between PSI and PSII, assuming that excitation energy is evenly distributed between the two photosystems.

ETR-I curves were fitted with the equation of Eilers and Peeters to estimate the photosynthetic parameters [32], _rel_ETR_max_ (maximal relative rate of linear electron transport), α (maximum light use efficiency which is the slope of the beginning of the light curve), and E*k* (light intensity for reaching _rel_ETR_max_).

For the non-photochemical quenching (NPQ) estimation, dark-adapted cells were illuminated with an actinic light setup at 480 µmol photons m^−2^ s^−1^ during 10 minutes, and the maximum fluorescence yield was estimated every min. NPQ was quantified by the Stern-Volmer expression: NPQ = (Fm/Fm′)–1.

### Pigments

Pigment measurement was conducted by High Performance Liquid Chromatography (HPLC). An aliquot of algal culture (10 mL) was taken with a pipette, immediately filtered (under low light condition) on 25 mm GF/F glass-fiber filter (Whatman, Maidstone, UK) and stored in liquid nitrogen until further analysis. Pigments were extracted by mechanical grounding during 3 minutes in 2 mL of a 100% methanol solution. Successively, the homogenate was filtered onto Whatman 25 mm GF/F glass-fiber filters and the volume of the extract was accurately measured. Prior to injection into the HPLC, 250 µL of an Ion Pairing Agent (ammonium acetate 1 mol L^–1^, final concentration 0.33 mol L^–1^) were added to 0.5 mL of the pigment extract and incubated for 5 minutes in darkness at 4°C. This extract was then injected in the 50 µL loop of the Hewlett Packard series 1100 HPLC (Hewlett Packard, Wilmington, NC, USA), equipped with a reversed-phase column (2.6 µm diameter C8 Kinetex column; 50 mm×4.6 mm; Phenomenex®, USA). The temperature of the column was steadily maintained at 20°C, and the flow rate of the mobile phase was set up at 1.7 mL min^–1^. The mobile phase was composed of two solvents mixture: A, methanol/aqueous ammonium acetate (70/30, v/v) and B, methanol. During the 12-minutes elution, the gradient between the solvents was programmed: 75% A (0 min), 50% A (1 min), 0% A (8 min), 0% A (11 min), 75% A (12 min). Pigments were detected spectrophotometrically at 440 nm using a Hewlett Packard photodiode array detector, model DAD series 1100. Fluorescent pigments were detected using a Hewlett Packard standard FLD cell series 1100 with excitation and emission wavelengths set at 407 nm and 665 nm, respectively. Determination and quantification of pigments were carried out using pigment standards from the D.H.I. Water & Environment (Horsholm, Denmark).

### Particulate Organic Carbon and Nitrogen

Ten mL aliquots for the determination of particulate organic carbon (POC) and particulate organic nitrogen (PON) were filtered on pre-combusted (450°C, 5 hours) glass-fiber filters (Whatman, Maidstone, UK), conserved in cell culture plates (Corning®, Corning Inc., NY, USA), and immediately stored at –20°C. The analyses were performed with a Thermo Scientific Flash EA 1112 automatic elemental analyzer (Thermo Fisher Scientific, MA, USA), following the procedure previously described by Hedges and Stern, [33]. Filters were thawed just prior to analysis and allowed to dry at 60°C through a desiccator. Then filters were loaded in small tin cups that were crimped closed and transferred to the CHN analyzer. A set of empty filters was processed as ordinary samples to accomplish the blank determination. Cyclohexanone 2,4-dinitrophenylhydrazone (C% 51.79, N% 20.14, H% 5.07) was used as standard.

### Absorption Spectrum

The spectral absorption measurements were performed using a spectrophotometer Hewlett Packard HP-8453E equipped with an inverted Labsphere integrating sphere (RSA-HP-53 Reflectance Spectroscopy Accessory). Ten mL aliquot was used to measure the spectral values of absorption coefficient (m^–1^) by intact cells [34]. Filtered cultures were used as references and the measurements were done in cuvette with 5 cm light path. The *a*(λ) values were measured between 250 nm to 800 nm, and integrated between 400 and 700 nm. This integrated value was divided by cell concentration for the estimation of the cell-specific absorption coefficient, a*, expressed in m^2^ cell^–1^.

The photosynthetically usuable radiation (PUR) was calculated using the following equation (Morel et al. [34]):
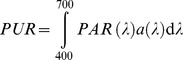



### Statistical Analysis

Student’s *t*-test and Spearman’s rank correlation was performed using Systat 7 software.

## Results and Discussion

### Spectral Radiations and Photoprotective Responses

**Figure 4:**
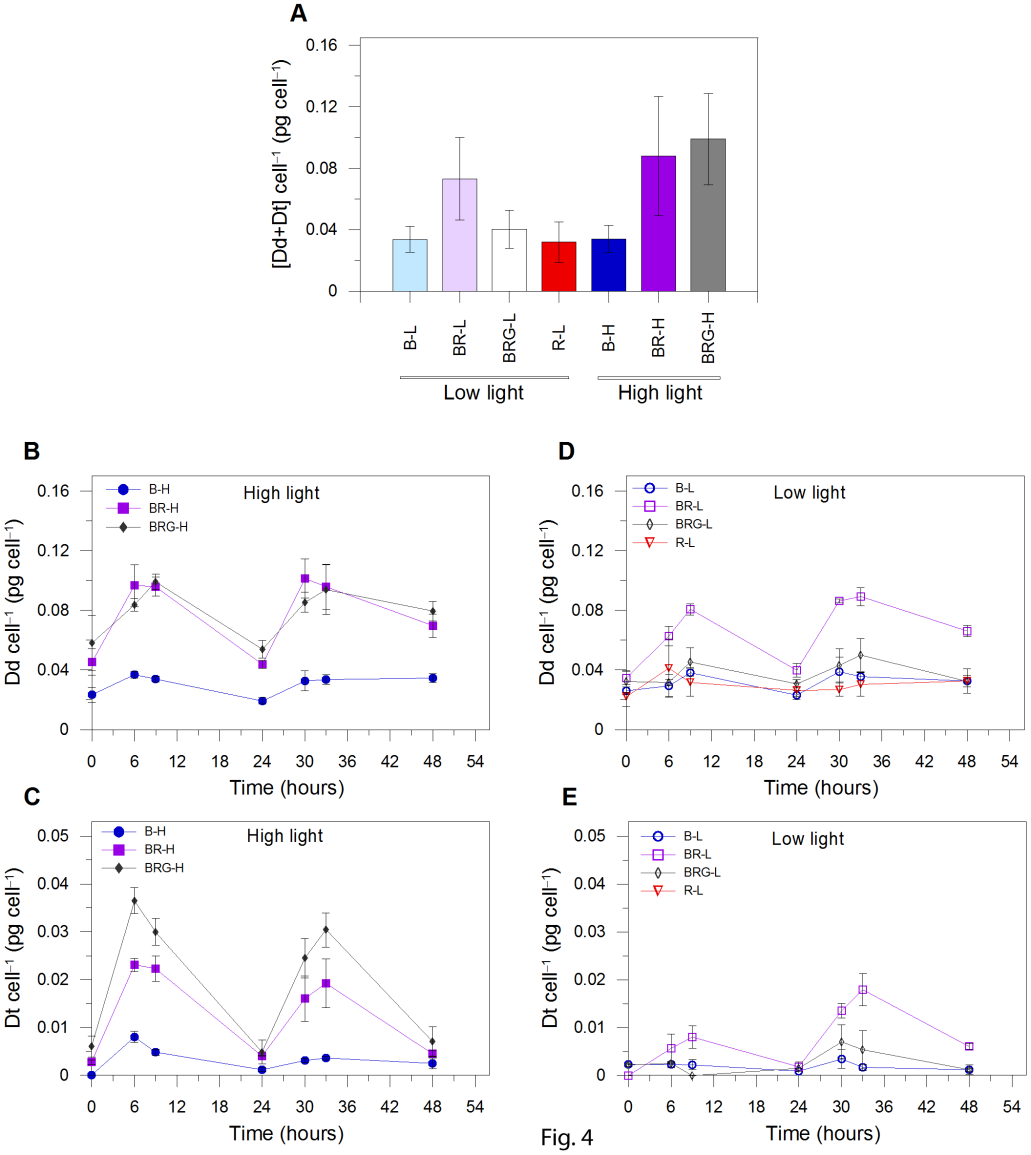
Variations of xanthophyll cycle pigment content. (A) sum of diadinoxanthin and diatoxanthin ([Dd+Dt]; pg cell^–1^); diadinoxanthin time distribution (Dd; pg cell^–1^) in high (B) and low light (D); diatoxanthin time distribution (Dt; pg cell^–1^) in high (C) and low light (E). B-L, BR-L, BRG-L, R-L are blue, blue-red, blue-red-green, and red low light conditions, respectively; B-H, BR-H, and BRG-H are blue, blue-red, blue-red-green high light conditions, respectively. Dt content was below detectable level in R-L. Time is in hours after the start of the experiment. Data represent (A) mean ± SD (*n* = 21) and (B–E) mean ± SD (*n* = 3).

The synthesis of xanthophyll cycle (XC) pigments, diadinoxanthin (Dd) and diatoxanthin (Dt), is higher under high light than low light (Fig. 4A, *p*<0.01, *n* = 21), with the exception of blue high light condition (B-H), in which cells did not increase the XC pigment pool. The low synthesis of both Dd and Dt under B-H (Fig. 4B,C) is not related to a variation in light absorption, since the absorption coefficient (a*, Table 1) and photosynthetically usable radiation (PUR, Table 1) in B-H were similar to the values found in BRG-H (blue-red-green high; *p*>0.05, *n = *3), in which Dd and Dt were significantly produced.

Therefore, to explain the absence of Dt and Dd synthesis in B-H, compared to BR-H (blue-red high) and BRG-H, we propose that the XC pigment synthesis in diatoms might require sensing of red light to be triggered, as well as the activation of blue-photoreceptors by high blue fluence rate to be activated [21]. Red light might act as a signal for cells to initiate the high light regulatory pathway, the intensity of the photoprotective response being thus determined by the blue fluence rate perceived by cells. This hypothesis fits with the results obtained under low light. Indeed, red radiation alone prevented Dt synthesis, and blue radiation alone was not able to enhance the Dt synthesis (Fig. 4A,D,E). Furthermore, significantly higher XC pigment content (Dd and Dt; Fig. 4A,D,E) was found in the BR-L (blue-red low) condition compared to B-L (blue low) and BRG-L (blue-red-green low; *p*<0.01, *n = *21). The absence of such XC activation under BRG-L is related to the low blue fluence rate experienced by cells (105 µmol photons m^–2^ s^–1^, Table 1), compared to BR-L (200 µmol photons m^–2^ s^–1^) or to high light conditions (≥190 µmol photons m^–2^ s^–1^, Table 1). Behind this interpretation, we know that green light induces much less effect on photoregulative processes than blue light in diatoms, which is in agreement with the lower PUR values measured in BRG (blue-red-green) compared to B (blue) and BR (blue-red) conditions (Table 1). This assumption is also supported by the absence of green-absorbing rhodopsin genes in coastal diatoms [35], as well as the by higher photosynthetic pigments content measured under green compared to blue light (data not shown), and by the fact that the pigments absorbing in blue-green region, Fuco and β-Car, were similar under BRG-L and BRG-H (see discussion below) and their distribution perfectly followed the Chl *a* content.

**Figure 5:**
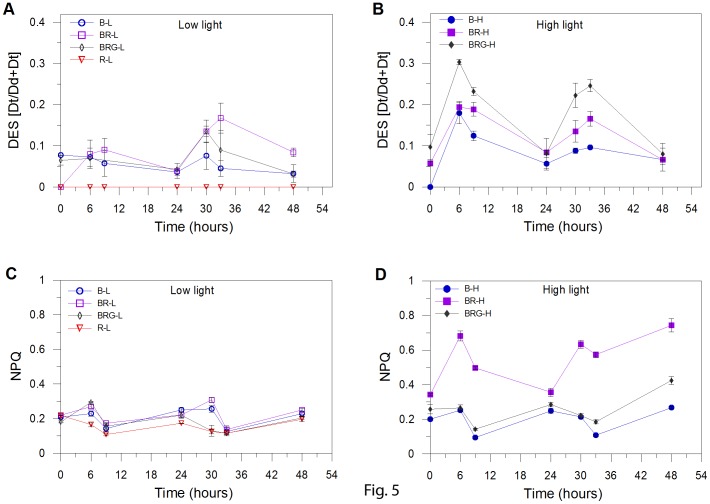
De-epoxidation state (DES = Dt/(Dd+Dt)) and non-photochemical quenching (NPQ). Time distribution of DES in (A) low and (B) high light. Time distribution of NPQ in low (C) and high light (D). B-L, BR-L, BRG-L, R-L are blue, blue-red, blue-red-green, and red low light conditions, respectively; B-H, BR-H, and BRG-H are blue, blue-red, blue-red-green high light conditions, respectively. Time is in hours after the start of the experiment. Data represent mean ± SD (*n* = 3).

The de-epoxidation state (DES), i.e. the Dd de-epoxidation into Dt, instead seems to be mainly up regulated by blue fluence rate as indicated by the higher DES under high than low light (Fig. 5A,B). This is found even when the synthesis of Dd and Dt is low, i.e. when red light is absent as in B-H. This feature reveals that Dd de-epoxidation does not depend on XC activation by the presence of both red and blue lights together; being enhanced by high blue fluence rate, as also observed by Schellenberger Costa *et al*. [21]. It would mean that the high light dependent-transthylakoidal **Δ**pH build-up [12], which activates the Dd de-epoxidase enzyme for transforming Dd into Dt, is not under control of the red perception signal. By contrast, the requirement of red light for enhancing both the Dd and Dt pigments would indicate that at least one of the enzymes involved into the XC photoprotective pathway [19] is under control of the red perception signal. By consequence, the Dd de-epoxidation rate being up regulated by the XC pigment content (Fig. 4A–E, 5A,B), is therefore dependent on the combination of red and blue radiations.

The NPQ capacity was enhanced only in BR-H (Fig. 5C,D), where the strongest NPQ was measured (0.66±0.13 at light peak, *n = *6). Intriguingly, the highest blue fluence rate (B-H) prevented NPQ increase, suggesting that NPQ development, as XC, required red light concomitantly with high blue fluence rate. The higher NPQ in BR-H than in BRG-H (0.25±0.03 at light peak, *n = *6; Fig. 5D) is due to the higher blue fluence rate experienced by cells (360 vs 189 µmol photons m^–2^ s^–1^, Table 1). In BRG-H, NPQ capacity was as low as the values obtained in B-H (no red radiation) and under low light (*p*>0.05, *n* = 21; Fig. 5C,D), despite the highest XC pool size and DES (Fig. 4A, 5B). Therefore, the NPQ development (Fig. 5C,D) is uncoupled with both the XC pool size (Fig. 4A) and DES (Fig. 5A,B), as also reported by Schellenberger Costa *et al*. [21] on *Phaeodactylum tricornutum* grown under different spectral light conditions. This uncoupling between XC and NPQ in BRG-H can be related to a weak functional activation of Dt molecules [10], [36] and to the heterogeneous spatial localization of Dt cellular pools [37]–[39]. Furthermore, the BRG-H condition, and the high red : blue ratio (0.43), might be a potential source of peroxidative damages in cells, that photoprotective xanthophylls can counter, as already observed in diatoms [8], [39].

### Spectral Radiations and Photosynthetic Pigment Content

**Figure 6:**
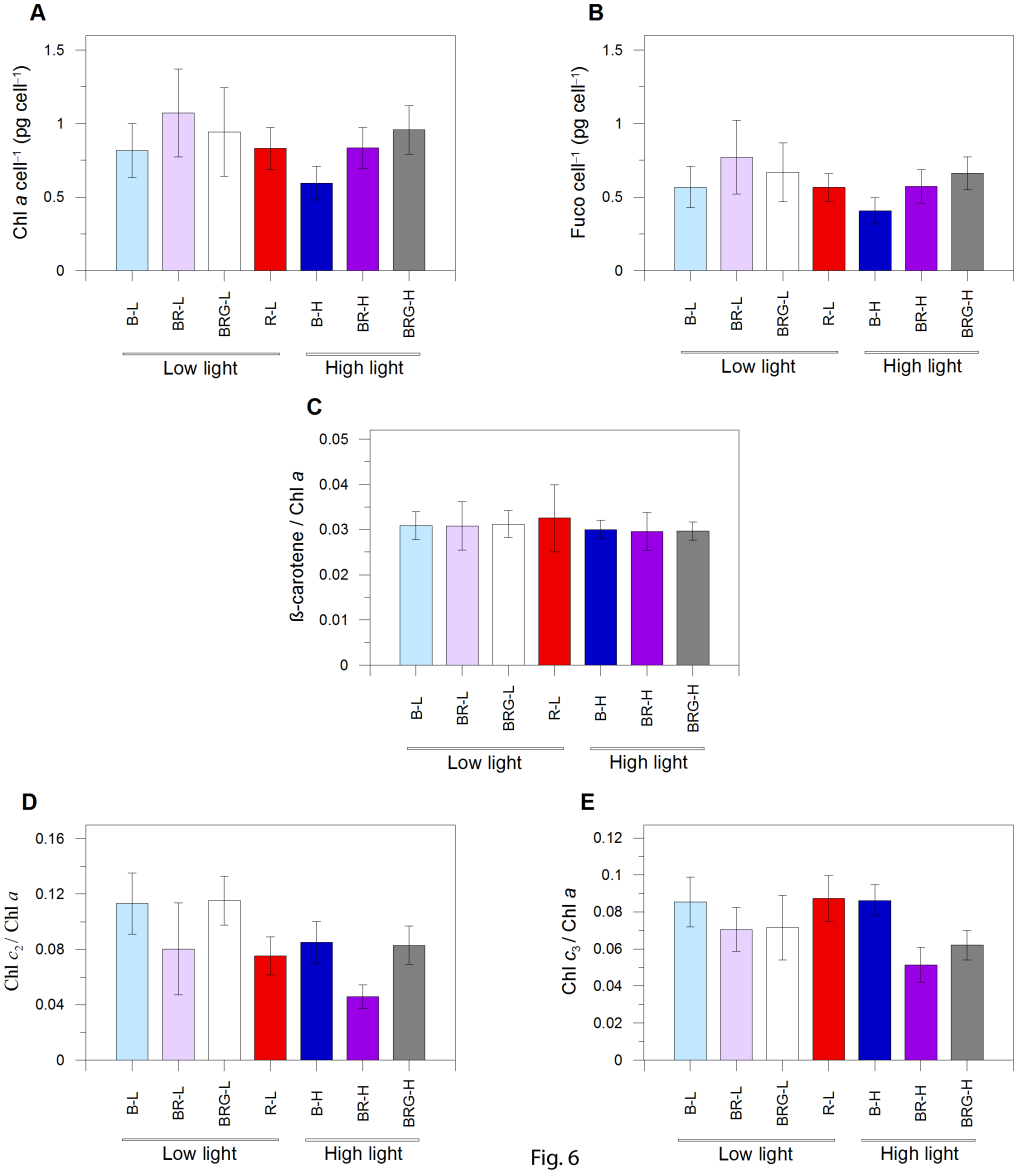
Variations of photosynthetic pigments content. (A) Chlorophyll *a* (Chl *a*; pg cell^–1^), (B) Fucoxanthin (Fuco; pg cell^–1^), (C) β-carotene : Chl *a* ratio, (D) Chlorophyll *c*_2_ : Chl *a* ratio and (E) Chlorophyll *c*_3_ : Chl *a* ratio. B-L, BR-L, BRG-L, R-L are blue, blue-red, blue-red-green, and red low light conditions, respectively; B-H, BR-H, and BRG-H are blue, blue-red, blue-red-green high light conditions, respectively. Data represent mean ± SD (*n* = 21).

Among the photosynthetic pigments, chlorophyll *a* (Chl *a*), fucoxanthin (Fuco) and β-Carotene (β-Car) followed the same trend over light conditions (Fig. 6A–C), with a stable ratio between those pigments (β-Car/Chl *a*, Fig. 6C; Fuco/Chl *a* : ≈ 0.70, data not shown). The contents of these three pigment decreased in B-H and BR-H compared to B-L and BR-L (Fig. 6A–C) as expected in a highlight photoacclimation state. The absence of such feature in BRG-H, where cellular pigment content was similar to BRG-L (Fig. 6A–C) can be relied to the lower blue fluence rate (189 µmol photons m^–2^ s^–1^ at the light peak) than in B-H and BR-H (≥360 µmol photons m^–2^ s^–1^, Table 1). This statement reinforces the strongest role of blue light on photosynthetic regulation in this diatom compared to green light (see discussion above) and coincides with the lower PUR values measured in BRG compared to B and BR conditions (*p*<0.05, *n* = 3; Table 1).

The co-variation of Fuco and β-Car with Chl *a* indicates a decreasing number of PSII units under high light, reveals an n-type photoacclimation strategy operated by the coastal diatom *P. multistriata*. This strategy allows to co-regulate the number of antennae and photosystem core complexes to finely tune the amount of absorbed light energy with the biochemical capacity of the cell. This is in agreement with the statement of Six et al. [40] and Lepetit et al. [9], who reported similar photoacclimation strategy by species growing in the upper mixed layer where light is variable.

Furthermore, our results reveal that, the high light-induced pigment variations do not require red radiation to be operated, on the contrary to XC activation or NPQ. This uncoupling between pigment variation occurring in the light harvesting complexes and the photoprotective XC activation, also fits with the absence of a significant relationship between Dd+Dt and Chl *a* or Fuco (*p*>0.05, *n* = 147). The reason might be linked to the different Dd content that the fucoxanthin chlorophyll a/c-binding protein (FCP) complexes might bind [41], [42]. Indeed, these two studies on two different diatoms revealed that two types of FCPs are present in diatoms, with different content of Dd, and that high light FCPs accommodate more Dd compared to low light FCPs.

Intriguingly, the two other accessory photosynthetic pigments, Chl *c*_2_ and *c*_3_ (Fig. 6D,E), showed unrelated variations with Fuco, β-Car and Chl *a* (Fig. 6A–C), varying both with PFD (decreasing under high light) and spectral conditions. Chl *c*_2/_Chl *a* ratio decreased under only red or low red : blue ratio conditions, as revealed by the significant lower value of this ratio in BR-L and R-L (red low) among low light conditions (*p*<0.05, *n* = 21) and in BR-H among high light conditions (*p*<0.05, *n* = 21). A concomitant effect of the low red : blue ratio on the decrease of Chl *c*_2_/Chl *a* and NPQ enhancement is observed, as also indicated by the significant correlation between Chl *c*_2/_Chl *a* ratio and NPQ (*p*<0.01, *n* = 147, data not shown).

Therefore, Chl *c*_2_ content in the FCP complexes can be preferentially modulated by light instead of Chl *a* and Fuco content. This result agrees with studies showing independent changes between Chl *c*_2_ and Fuco content (e.g., in *Pelagomonas calceolata*, Dimier et al. [43] and in *Phaeodactylum tricornutum*, see Fig. 6 in Nymark et al. [44]). Indeed recently Gundermann et al. [45] showed that FCPs mainly exist as trimers in *P. tricornutum* and sub-fractioning of FCP complexes from low and high light, yielded different populations of trimeric complexes. Under low light, the trimers mainly containing Lhcf5 proteins were characterised by low Fuco : Chl *c* ratio while under high light Lhcf5 was significantly reduced and trimers containing Lhcf4 proteins were characterised by high Fuco : Chl *c* ratio. From both the Gundermann et al. [45] study and our results, we can hypothesize that the high light regulation of Lhcf5 *vs* Lhcf4 proteins content requires red radiation and high blue fluence rate (i.e., under low red : blue ratio).

Even though the absorption properties of Chl *c*_3_ and Chl *c*_2_ are almost similar, Chl *c*_3/_Chl *a* varied irrespectively to the red : blue ratio value (Fig. 6E). The monospectral light conditions (blue or red), presented higher and similar Chl *c*_3/_Chl *a* ratio (Fig. 6E), while this ratio similarly decreased in the two mixed light conditions (BR and BRG) under low and high light (*p*<0.05, *n* = 21). *Pseudo-nitszchia multistriata* is one of the few diatoms presenting Chl *c*_3_ pigment [46]. The spectral radiation modulation of Chl *c*_3_ content, decreasing when both red and blue radiations are present together, fits with the increase of Chl *c*_3_ observed in the deep, layer below 50 metres depth in the Mediterranean Sea, where only blue light is present and red light is absent [47]. In contrast to Chl *c*_2_, little information is available on the Chl *c*_3_ pigment mainly because this pigment is rarely found in diatoms, but mostly in haptophytes [48] and pelagophytes [43].

### Spectral Radiations and Growth and Cell Properties

When provided with low PFD, red light induces a significant decrease of growth rate compared to the other conditions (Fig. 3A,B). Recently, Schellenberger Costa et al. [21] showed on *Phaedoactylum tricornutum* that usage of light energy was less efficient when cells were grown under red light as to blue or white light. The low growth capacity in red condition is related to an undergoing physiological stress, which also could explain the growth inhibition under high red light (data not shown), as revealed by the significantly higher POC content (*p*<0.05, *n* = 21; Table 1) together with the lower particulate organic carbon (POC) : particulate organic nitrogen (PON) ratio (*p*<0.01, *n* = 21; Table 1) and chlorophyll *a* : POC ratio (*p*<0.05, *n* = 21; Table 1). Interestingly, in all conditions with blue light, cells achieve almost similar growth (Fig. 3A,B), revealing an efficient development of acclimative features to the different conditions, by varying their energy allocation strategies or the arrangement of biochemical pathways [49].

The fastest cell number increase during the exponential growth phase recorded under B-H (Fig. 3B) is paralleled by a low POC/PON ratio (Table 1), in agreement with the findings of Halsey et al. [49]. The lack of red-dependent activation of the xanthophyll cycle and non-photochemical quenching in B-H, limiting the energetic cost of the photoprotective response [43], allows therefore a highest energetic investment for growth. In turn, cells being unable to balance light harvesting and photoprotective capacity, are not able to cope with high light damages, leading cells earlier into stationary phase under this condition (B-H) compared to other high light conditions.

The low red : blue ratio (0.25, BR-H and BR-L) appears to be peculiar, when compared to the other light conditions, since growth capacity (Fig. 3), absorption coefficient (a*), as well as photosynthetic properties and POC and PON content (Table 1) are similar between low and high PFD. At the exception of R-L, the highest a* is found in BR (Table 1, *p*<0.001, *n* = 3) and is paralleled by the enhancement of the maximal relative rate of linear electron transport (_rel_ETR_max_), light intensity for reaching the _rel_ETR_max_ (E*k*) and the maximum light use efficiency under low light (α), thus revealing relevant changes of the photosynthetic properties under this condition (Table 1), irrespective of PFD experienced. Moreover, as consequence of the highest PUR in BR-H, which results in an excess energy absorption, cells undergo an efficient photoprotection, by increasing Dt content (Fig. 4) and by developing high NPQ (Fig. 5D). The causes of the high a* in BR-H, similar to BR-L (Table 1), and therefore the highest PUR in BR-H, are unclear, not being explained by significant variations in pigment content (Fig. 6A–C). The uncoupling between pigment variation and a* in BR-H might concern variations in pigment package effect. The latter can be induced by structural changes in thylakoid membranes [50], [51], occurring in BR-H as revealed by the variations in Chl *c* content, and by the changes in LHCs properties (see discussion above, Gundermann et al. [45]).

## Conclusion

Our study leads to consider the spectral composition of light as an essential trigger for photophysiological acclimation of diatoms. Our results suggest that the fast photoprotective processes such as XC and NPQ require red light to be initiated and a high blue fluence rate to be activated. These results logically fit with the optical properties of the water column, since red radiation is only present in the upper layer of the water column, i.e. associated to high PFD (Fig. 1; [5]). Hypothetically, red radiation sensed by cells in the surface layer, act as a relevant environmental cue [20] for signalling high light environment, while blue fluence rate experienced by cells, narrowly correlated to the depth at which cells are upwelled, determines the strength of the photoprotective XC activation, NPQ development and pigment content variations. Furthermore, the red : blue ratio is also a crucial parameter for shaping photophysiological properties of the cells, mainly linked to pigment content related to light-harvesting complex structures.
